# The Effect of Prefabricated vs Custom Fiber-Reinforced Posts on the Push-Out Bond Strength of Self-Adhesive Resin Cement in Simulated Flared Root Canals: An in Vitro Study

**DOI:** 10.3390/polym18212641

**Published:** 2026-10-25

**Authors:** Maher S. Hajjaj, Lyan Y. Qari, Maram A. AlKenani, Anhar I. Basunbul, Dalea M. Bukhary, Abeer K. Tashkandi, Afnan O. Al-Zain, Hanin E. Yeslam

**Affiliations:** 1 Department of Restorative Dentistry, Faculty of Dentistry, King Abdulaziz University, Jeddah 21589, Saudi Arabia; btashkndee@kau.edu.sa (A.K.T.); alzain@kau.edu.sa (A.O.A.-Z.); ayaslam@kau.edu.sa (H.E.Y.); 2 Advanced Technology Dental Research Laboratory (ATDRL), Faculty of Dentistry, King Abdulaziz University, P.O. Box 80209, Jeddah 21589, Saudi Arabia; 3 Department of Cariology, Restorative Sciences, and Endodontics, School of Dentistry, University of Michigan, Ann Arbor, MI 48109, USA; lqari@umich.edu; 4 Saudi Board Residency Program, Department of Pediatric Dentistry, King Abdullah Medical Complex, Saudi Commission for Health Specialties (SCFHS), Jeddah 11614, Saudi Arabia; maramaalke@gmail.com; 5 Oral and Maxillofacial Prosthodontics Department, Faculty of Dentistry, King Abdulaziz University, Jeddah 21589, Saudi Arabia; aabasunbol@kau.edu.sa (A.I.B.); dbukhary@kau.edu.sa (D.M.B.)

**Keywords:** push-out bond strength, different post designs, 3DP, flared root canal, CAD/CAM, in vitro study

## Abstract

Objectives: In this study, we aimed to evaluate the effect of post design on the push-out bond strength (PBS) of self-adhesive resin cement in simulated flared root canals. Materials and Methods: A total of 135 specimens simulating flared single root canals were fabricated using standardized 3D-printed resin (FREEPRINT^®^ CROWN; Detax GmbH, Ettlingen, Germany) and allocated into three groups: single, double, and customized glass fiber-reinforced posts (Pillar Fiber Post, TiaDent, TX, USA). All specimens were cemented using self-adhesive resin cement (Rely X^TM^ U200, 3M ESPE, MN, USA). The specimens were then sectioned perpendicular to the long axis into coronal, middle, and apical slices, each measuring 3 mm. After thermocycling, they were tested for their push-out bond strength. Failure modes were analyzed using stereomicroscopy. A statistical analysis was completed for PBS data using a two-way ANOVA test, and failure modes were analyzed using a Chi-square test at a significance level of α = 0.05. Results: We observed statistically significant differences in the PBS between test groups (*p* = 0.044*). Double-post specimens exhibited the highest PBS values at the coronal section (8.26 ± 1.54 MPa), followed by the middle section (7.77 ± 1.73 MPa). Across all root sections, the PBS of double posts (7.58 ± 2.07 MPa) significantly outperformed customized (6.76 ± 1.15 MPa) and single-post (6.73 ± 1.75 MPa) groups (*p* = 0.029*). The coronal third (7.44 ± 1.54 MPa) consistently showed higher PBS compared to the middle (7.15 ± 1.64 MPa) and apical (6.49 ± 1.90 MPa) thirds (*p* = 0.027*). Failure mode analysis revealed that customized posts predominantly failed at the post-adhesive interface, whereas double posts showed a mix of adhesive failures and single posts demonstrated more adhesive failures at the canal walls. Conclusions: The use of double fiber posts significantly enhances push-out bond strength in flared canals compared to single or customized posts. Relining techniques improved canal adaptation, but the performance here did not surpass that of double-post configurations. Clinically, the double-post approach may present a simple, practical and effective alternative to improve the bond strength of fiber-reinforced posts for flared root canal systems.

## 1. Introduction

Restoring endodontically treated teeth (ETTs) presents a daily challenge in the clinical setting, with these teeth being more susceptible to tissue fracture after the placement of the final restoration [1,2,3,4,5,6]. In such cases, it is important to retain the final restoration using an intraradicular post system with the appropriate distribution of stresses throughout the remaining tooth structure. Glass fiber-reinforced posts (FRPs) have gained popularity over metallic posts due to their favorable elastic modulus, which is closer to that of dentin, allowing for better stress distribution and a reduced risk of root fracture. Additionally, they offer esthetic advantages, can be completed in a single visit, and are compatible with adhesive systems, making them suitable for use in esthetic practices [7,8,9,10,11]. However, despite these benefits, one of the most commonly reported causes of failure for fiber post restorations is debonding [7,8,12,13,14]. Difficulties arise from the inability of prefabricated posts to adapt closely to irregular canal walls, resulting in thick resin cement layers that are prone to polymerization shrinkage and void formation. This mismatch between the post and canal morphology is recognized as a major cause of failure for fiber post restorations [7,8,15,16].

To overcome this issue, several techniques have been investigated, aiming to improve post adaptation and minimize the thickness of the cement layer. Among these, the anatomical customization of posts using composite resin has shown promising results in improving retention [8,12,17,18]. Such studies explored the concept of relining fiber-reinforced posts with composite resin to reduce the cement film thickness, thus increasing adaptability and, consequently, the push-out bond strength [7,8,12,19]. Other studies have explored using accessory post systems to reinforce the main post [15,20]. However, there is disagreement in the literature regarding the effectiveness of such approaches. For instance, Latempa et al. [15] used accessory posts that significantly improved the push-out bond strength (PBS) in flared canals. In contrast, Silva et al. [12] found no significant difference in PBS between relined and non-relined posts under certain conditions. Although customized and relined posts have been widely explored in the literature, fewer studies have evaluated flared canal restoration using a dual-post system with prefabricated posts of different sizes. Dual and multiple posts have been proposed to enhance retention by increasing frictional contact and reducing shrinkage stresses [16,20,21,22]. However, methodological challenges have been reported in laboratory studies involving severely flared canals. For example, Li et al. [16] noted difficulty in standardizing extensively destroyed root canals in a laboratory setting, necessitating artificial coronal enlargement and predefined ferrule dimensions, which may have contributed to unconventional results regarding pull-out retention; in contrast, the standardized canal preparation and specimen selection adopted in the present study were intended to minimize such variability and allow a more reliable comparison of post retention.

When testing PBS, natural teeth are usually considered the best option for simulating the clinical condition [12,15,23,24,25,26]. Despite this, natural teeth are adversely impacted by the fact that each tooth has its own history pertaining to the patient’s age, the date of extraction, the degree of calcification, and even the amount of moisture. During sampling, microcracks, inherent wear, defects, and varying root canal configurations can often lead to large variations in results [16,27,28,29,30], which may negatively affect the bonding assessment [31]. This study introduces a new approach to evaluate the bond strength of FRPs using three-dimensionally printed (3DP) resin dies simulating flared root canals. The PBS values of three different FRP designs (single, double, and custom) were evaluated at three different levels (coronal, middle, and apical). To the best of the authors’ knowledge, this study design has yet to be evaluated in the literature. Our research questions were as follows: First, does the use of customized vs prefabricated (single or double) FRPs improve the push-out bond strength for 3DP root canals? Second, is there a significant difference in the push-out strength at different levels (coronal, middle, and apical) of the canal space? Third, what is the effect of different FRP designs on the failure mode at different canal space levels? The null hypothesis declares no significant difference in the push-out bond strength and failure modes across post designs and root canal levels.

## 2. Materials and Methods

### 2.1. Sample Size Calculation

The G*Power 3.1.9.7 statistical software was used to compute the required sample size. The effect size (f) was set to 0.4 at a significance level of α = 0.05 and power of 80%, with 9 test groups. The computed total sample size was 108 specimens; dividing this value by 9 (the number of test groups) led to 12 specimens/group. To overcome any sampling errors, 15 specimens/group were fabricated, with a total sample size of 135 specimens.

### 2.2. 3DP Model Fabrication

**Figure 1:**
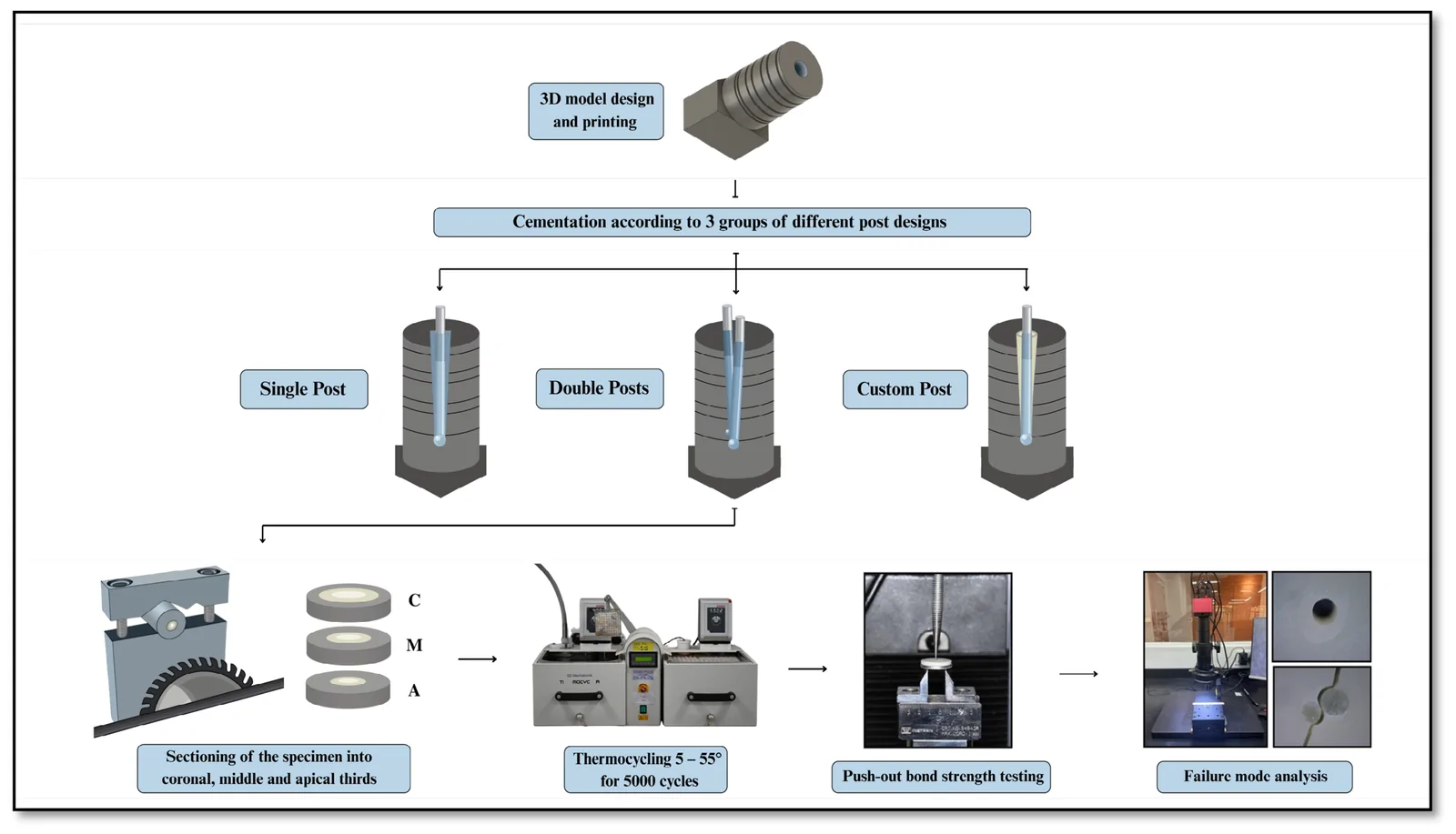
Schematic illustration of the experimental workflow used in the study.

3D models of simulated flared root canals (coronal dimension of 4 × 3 mm and an apical diameter of 1.5 mm) were generated in the Autodesk Fusion 360 software (version 2.0.21550, Autodesk Inc., San Francisco, CA, USA). Parametric modeling allowed us to achieve consistent internal geometries across all test specimens. The chosen dimensions were modeled to simulate severe root canal flaring, commonly resulting from over-instrumentation, extensive endodontic access, trauma, or internal root resorption. These dimensions directly reflect the anatomical dimensions established in prior ex vivo studies utilizing extracted human teeth [16,32,33]. Grooves on the models marked areas of desired cutting, ensuring a standardized, 3 mm thick disk that was uniformly perpendicular to the long axis of the model. Finally, to ensure standardization and ease of cutting, a base was fabricated, exactly matching the dimensions of the saddle clamp to be used during cutting. This base ensured the models were fixed at a 90° angle to the saw machine, guaranteeing 3 mm cuts throughout the thickness of each disk (Figure 1).

Resin specimens were 3D-printed via digital light processing (DLP) technology using commercially available light-cured resin (FREEPRINT^®^ CROWN; Detax, Ettlingen, Germany), intended for use in single, permanent indirect restorations, long-term provisional bridges, and denture teeth [34,35]. This resin was selected because of its adequate mechanical properties and good bond strength for self-adhesive resin cement [36]. Forty-five models (135 specimens divided by 3 specimens/model = 45 models) were printed using an AccuFab-L4D printer (Shining 3D, Hangzhou, China). The printing parameters included a layer thickness of 50 μm, a print speed of 40 mm/h, and a build temperature of 25 °C. After that, the printed specimens were disconnected from their supports and cleaned with 98% isopropyl alcohol for 3 min; then, the post space was thoroughly cleaned with compressed air. A second cleaning cycle was performed in a separate vessel with fresh isopropyl alcohol for 2 min. Prior to curing, specimens were inspected for defects or excess resin and dried off using compressed air. For post-curing of the printed models, a UV curing unit (FabCure; Shining 3D, Hangzhou, China) was used at a wavelength of 405 nm, with a curing duration of 30 min, as per the manufacturer’s instructions.

### 2.3. Post-Cementation

The 3DP models were randomly divided into three groups based on the post design (n = 15): group 1 utilized a single FRP, group 2 utilized double FRPs, and group 3 utilized a customized FRP relined with a composite. Post spaces were irrigated with normal saline and dried with paper points (Meta Biomed Paper Points; Meta Biomed Co., Ltd., Cheongju, Korea). In group 1, the post space was filled with self-adhesive resin cement (SARC) (RelyX U200 Automix; 3M ESPE, Seefeld, Germany), and size #3 FRPs (Pillar Fiber Post; Tiadent inc., Rosenberg, TX, USA) were inserted to the full working length, followed by light polymerization through the post for 40 s (Bluephase N Cure; Ivoclar Vivadent, Schaan, Liechtenstein) at 1200 mW/cm^2^. For group 2, after filling the post space with SARC, a size #3 post was inserted to the full working length; then, an accessory post of size #2 was inserted to the maximum depth allowed, followed by light curing. For group 3, the post space was lubricated with water-based lubricant (Ultrasound Gel; Saudi Pharmaceutical Industries & Medical Appliances Co., Riyadh, Saudi Arabia). The posts then received one coat of a universal adhesive (Scotchbond Universal; 3M ESPE, St. Paul, MN, USA) and were light-activated for 10 s according to the manufacturer’s instructions. Next, a universal restorative composite (Filtek Z250; 3M ESPE, St. Paul, MN, USA) was molded on a size #3 post and inserted into the post space; it was then light-cured for 5 s, and the custom post was retrieved and light-cured for an additional 40 s. After that, the post space was thoroughly irrigated and dried and filled with SARC; then, the custom post was inserted and cured for 40 s. All specimens were stored in normal saline in a 37 °C incubator for 48 h. The composition and manufacturers of the materials used in the current study are summarized in Table 1.

### 2.4. Push-Out Bond Strength Test

Specimens were sectioned using a low-speed diamond saw (TechCut 4 Low-Speed Diamond Saw; Allied High-Tech Products Inc., Rancho Dominguez, CA, USA) under water cooling to obtain coronal, middle, and apical slices, each 3 mm thick, at the predetermined level. All specimens were subjected to an artificial aging process involving thermocycling at 5–55 °C for 5000 cycles in the SD Mechatronik Thermo-cycler (JulaBO GmbH, Seelbach, Germany). The push-out bond strength test was conducted using a universal testing machine (Instron, Norwood, MA, USA) in the apical-to-coronal direction at 0.5 mm/minute until the post was dislodged. The maximum load to failure was measured in Newtons and then converted to bond strength (MPa) by dividing the load by the surface area of the bonding area. The bonding surface area was calculated using the following formula:

$$A=\pi \left(R+r\right)\sqrt{h^{2}+{\left(R-r\right)}^{2}}$$

where R is the coronal radius, r is the apical radius of the post, and h is the thickness of the disk [37]. The bond strength was then calculated for each specimen:

$$\mathrm{Bond Strength}\left(\mathrm{MPa}\right)=\frac{\mathrm{Maximum Load}\;(N)}{A\;({\mathrm{mm}}^{2})}$$

### 2.5. Failure Mode Assessment

All samples were then analyzed using stereomicroscopy (RaySmart Technology Co., Ltd., Shenzhen, China) at a magnification of 60× to evaluate failure modes. Recorded failure modes were classified into the following categories: 1: adhesive failure between the cement and walls (canal walls were completely exposed with no cement); 2: adhesive failure between the cement and the post (canal walls were covered with cement); 3: mixed failure, where remnants of cement cover the canal walls; and 4: cohesive failure within the printed model.

### 2.6. Statistical Analyses

Statistical analysis was conducted using JMP Version 19 Statistical Discovery from SAS Software (JMP; SAS Campus Drive, Cary, NC, USA). Shapiro’s test of normality showed that PBS data maintained a normal distribution, allowing for parametric testing. A two-way ANOVA was performed to assess the effects of the fiber post design, the root section level, and their interaction (fiber post design * level of section) on the push-out bond strength of self-adhesive resin cement when used in 3DP flared canals. Post hoc pairwise comparisons were performed using the Tukey test. Additionally, a Chi-square test was employed to analyze the modes of failure. All statistical tests were conducted at a significance level of α = 0.05.

## 3. Results

### 3.1. Push-Out Bond Strength Analysis

A total of 135 specimens were analyzed. Descriptive statistics (mean, standard deviation, and two-way ANOVA) are summarized in Table 2. The two-way ANOVA test showed a statistically significant difference between test groups (*p* = 0.044*). Regarding the independent variables, both post design (*p* = 0.027*) and root section (*p* = 0.025*) significantly affected the push-out bond strength. However, the interaction factor of “post design*root section” was not significant (*p* = 0.823), indicating that their effects on PBS were independent of each other and did not introduce any additional variation beyond their individual contributions. 

**Figure 2:**
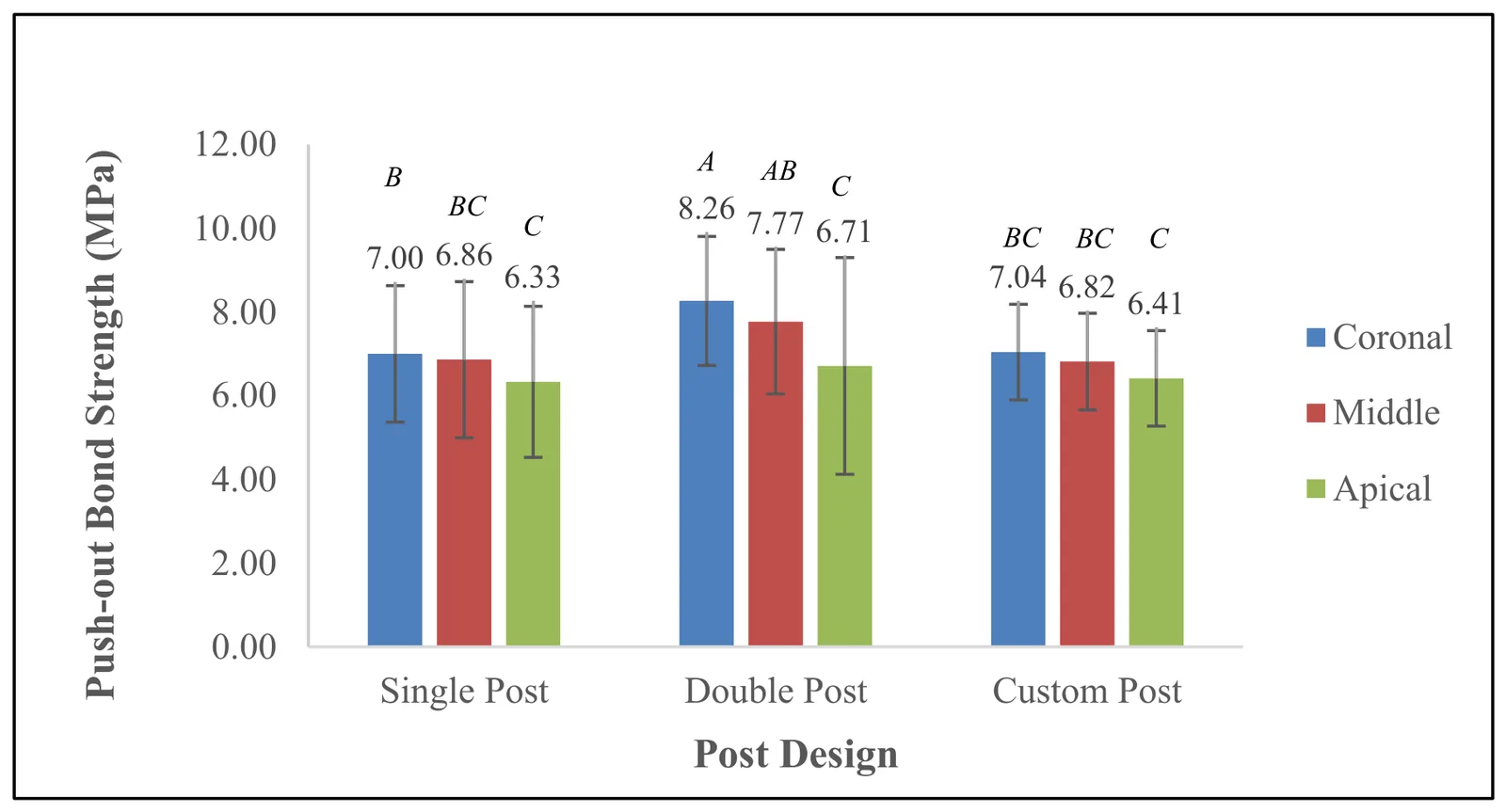
Push-out bond strength values according to post design and root section. Groups connected with similar letters indicate no statistically significant difference (*p* < 0.05).

The highest PBS was observed in the double-post design at the coronal section (8.26 ± 1.54 MPa), followed by the middle section (7.77 ± 1.73 MPa). The custom-post design exhibited lower bond strength values, with the coronal section measuring 7.04 ± 1.14 MPa, the middle section 6.82 ± 1.16 MPa, and the apical section 6.41 ± 1.14 MPa. The single-post design showed comparable values to the custom-post group, with the highest bond strength at the coronal section (7.00 ± 1.63 MPa), followed by the middle (6.86 ± 1.87 MPa) and apical (6.33 ± 1.81 MPa) sections. The middle third did not differ significantly from the coronal or apical thirds for the custom-post design (*p* > 0.05). However, the middle third (PBS = 7.77 ± 1.73 MPa) did significantly differ from the coronal third (PBS = 8.26 ± 1.54 MPa) for the double-post design, as well as for the single-post design (*p* = 0.025* and *p* = 0.027*, respectively). Additionally, the middle third was statistically different from the apical third for the double-post design (*p* = 0.029*) (Table 2, Figure 2).

Table 3 describes the estimated marginal means based on the independent variable of “post design”. This test evaluates the PBS values for each post design, averaged across all root sections. Double posts exhibited the highest PBS (7.58 ± 2.07 MPa), which was significantly greater than that of both custom posts (6.76 ± 1.15 MPa) and single posts (6.73 ± 1.75 MPa) (*p* = 0.029*).

Table 4 describes the estimated marginal means based on the independent variable of “root section”. This test evaluates the PBS for each section of the root, averaged across all post designs. Bond strength values significantly decreased from the coronal to apical thirds (*p* = 0.027*). The coronal section demonstrated the highest bond strength across all post designs (7.44 ± 1.54 MPa), statistically similar to the middle (7.15 ± 1.64 MPa) but significantly higher than the apical (6.49 ± 1.90 MPa) sections.

### 3.2. Failure Mode Analysis

**Figure 3:**
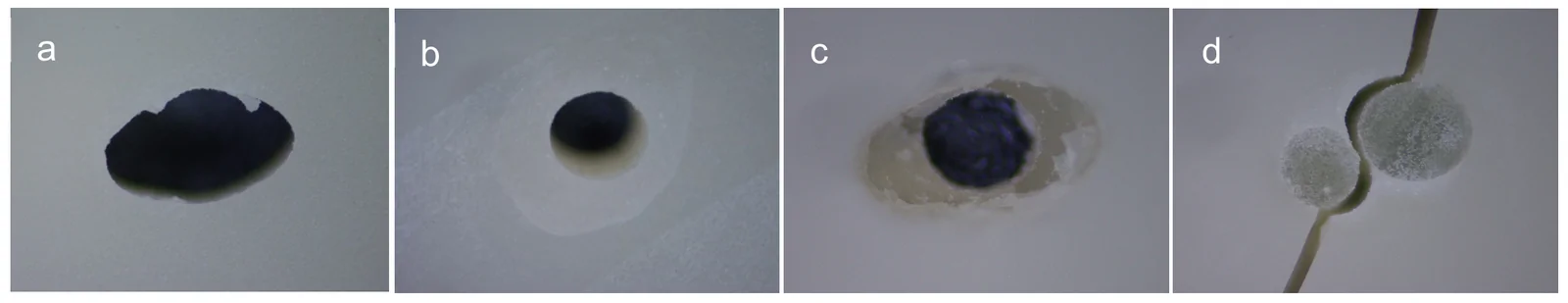
Failure mode analysis: (**a**) adhesive failure between cement and canal walls; (**b**) adhesive failure between cement and post; (**c**) mixed failure; and (**d**) cohesive failure within the model.

Failure mode evaluation was conducted on all 135 specimens using 60× stereomicroscopy. Observed failures were classified into four categories based on their cleanliness and composite distribution on the canal walls (Figure 3). The Chi-square test showed a significant difference in the failure mode distribution between test groups (*p* < 0.0001).

The custom-post group showed a clear predominance of mode 2 failures (adhesive failure between the post and cement/composite), particularly in the apical (80.00%) and middle sections (60.00%). Coronal sections of this group displayed more variation, with 40.00% in mode 2, 26.67% in mode 3, and 33.33% in mode 4.

In contrast, the double-post group demonstrated a high frequency of adhesive failures regarding the post (mode 2) and mixed adhesive failures (mode 3). The apical section exhibited 64.29% of mode 2 failures, while the middle section showed an even split among modes 2 (40.00%), 3 (40.00%), and 1 (13.33%). Notably, the coronal region had a high rate (60.00%) of mode 4 failures.

The single-post group was dominated by mode 1 (canal walls completely exposed) failures in the apical (46.67%) and middle sections (33.33%). The coronal section presented a high incidence (80.00%) of mode 2 failure (canal walls completely covered), reflecting weak bonding at the post interface (Table 5).

## 4. Discussion

Numerous studies have explored methods to enhance the retention of fiber posts in flared canals. However, the lack of standardized techniques has led to variability in outcomes [38,39,40]. In this study, we aimed to assess the push-out bond strength (PBS) and failure modes of simulated flared root canals restored with customized and prefabricated (single or double) fiber posts, using a standardized 3D-printed model and a uniform cementation protocol. Significant differences were observed in PBS and failure modes between test groups (*p* = 0.044*), so the null hypothesis was rejected. Both post design and root section significantly influenced the bond strength values and failure modes.

The results revealed that post design and root section both had statistically significant effects on PBS, with double posts demonstrating the highest bond strength followed in turn by custom and single posts. This pattern is consistent with earlier findings suggesting that increased adaptation and reduced cement thickness can significantly enhance interfacial bonding [7,41]. Several studies have reported that relining fiber posts improves bond strength due to the presence of thinner cement layers and reduced voids, limiting polymerization shrinkage and stress concentrations [12,42].

Frictional retention is also directly proportional to the contact area; the larger the contact surface, the better the retention. This explains the corono-apically declining bond strength values. Such a reduction in bond strength toward the apical third may be attributed to several factors. The high configuration factor (C-factor) in the root canal, particularly in the apical region, can lead to increased polymerization shrinkage stress, promoting gap formation and weakening the adhesive interface [43,44,45,46,47]. In addition, limited light transmission in deeper root sections may compromise the conversion degree of the dual-cure self-adhesive cement [48,49,50]. The reduced diameter and surface area in the apical third also provide less mechanical interlocking and retention, further contributing to the observed decrease in bond strength [38,51,52].

Nonetheless, the custom group in this study did not outperform the double-post group. This contrasts with the findings from Junior et al. [8], where relined glass fiber posts presented the highest push-out bond strength regardless of the resin cement used. This discrepancy may stem from the inadequate bond between the fiber post and relining composite. Mode 2 failures were observed more frequently in group C, with 80.00% and 60.00% in the apical and middle sections, respectively, showing that most of the composite and cement remained bonded to the walls of the canals and did not provide mechanical resistance during PBS testing. This finding highlights the importance of optimizing the interface between the fiber post and the relining composite. Future studies are warranted to explore methods that enhance this interface, such as modifying the surface of the fiber post through etching, sandblasting, or the use of serrated designs. Additionally, applying compatible bonding agents or using pre-silanated posts may improve adhesion and reinforce the mechanical integrity of the post–composite complex.

The double-fiber-post configuration exhibited the highest push-out bond strength, particularly in the coronal (8.26 ± 1.54 MPa) and middle (7.77 ± 1.73 MPa) root regions. This may be attributed to improved mechanical interlocking and a reduced resin cement volume. Similar outcomes were observed by Lopes et al., who demonstrated that better adaptation of the post, achieved either via relining or accessory post use, increased retention and minimized void formation within the cement layer [53]. In another study, Latempa et al. demonstrated that incorporating two accessory fiber posts alongside a main post significantly improved bond strength compared with smaller single-post configurations [15]. This technique potentially replicates the benefits of anatomical customization with less clinical complexity, offering a viable alternative when relining is not feasible. Conversely, other investigations have reported limited or unfavorable effects of accessory posts on bond strength, indicating that the mechanical benefits of multiple-post configurations may depend on the post geometry, cementation protocols, and experimental conditions [16,54]. Therefore, the superior bond strength observed in this study’s double-post group may reflect more favorable adaptation within the flared canal rather than an inherent advantage of increasing the number of posts. Despite these insights, the lack of interaction between post design and root section indicates their independent effects on PBS; regardless of the root level, post design consistently influences bonding behavior, a pattern observed in other studies using similar statistical models [55]. Using 3DP resin dies instead of natural teeth for PBS testing has several advantages: it allows high standardization of the canal diameter, taper, and length; reproducibility across specimens, reducing anatomical variability; control over the cement thickness; the elimination of anatomical confounders such as dentinal tubule density, canal irregularities, and moisture levels; and a reduced dependence on extracted teeth, limiting the difficulties involved in acquiring sufficient samples with comparable morphology and dimensions. Finally, digital STL files permit fabricating the same canal configuration repeatedly, improving experimental consistency. While this study standardized many variables through the use of 3D-printed root canal models to minimize anatomical variability, several limitations must be acknowledged. Printed resin does not reproduce the composition or bonding characteristics of root dentin. The difference in elastic modulus may affect stress distribution during PBS testing. Materials with a chemical composition closer to natural tooth tissue (bone substitutes) are worth exploring in future studies. An increased variability in PBS was observed in the apical region, particularly in the double-post group, suggesting that technique- and material-dependent factors—rather than root morphology—may contribute to this dispersion. In addition, the custom-post group exhibited a high percentage of failures between the composite and post (mode 2), suggesting a weak interface at this junction. The absence of surface modification of the post or silane application during the relining process may have contributed to this outcome. Future studies could investigate methods of enhancing the interfacial adhesion between composites and posts to reduce such failures. Finally, our results cannot be directly extrapolated to the clinical retention or long-term survival of fiber posts in human teeth.

Additionally, the in vitro nature of this study, while allowing for strict control over variables, does not fully replicate the complex oral environment, where factors such as cyclic loading, humidity, and thermal stresses continuously affect the integrity of post restorations. Furthermore, only stereomicroscopic images were evaluated; advanced imaging using scanning electron microscopy or micro-computed tomography would aid us in evaluating cement film thickness and adaptation. Another limitation is our use of only one type of self-adhesive resin cement and one composite material; results may vary with different resin cements, composite materials, or even serrated posts. Moreover, the push-out bond strength test primarily assesses resistance to dislodgement in a single direction and does not fully represent the multidirectional forces experienced clinically. Long-term fatigue testing and clinical evaluations are necessary to validate the findings observed under static conditions.

## 5. Conclusions

In this study, post design significantly influenced the push-out bond strength, with double-post configurations demonstrating the highest values (7.58 ± 2.07 MPa) followed by customized relined posts (6.76 ± 1.15 MPa) and finally single posts (6.73 ± 1.75 MPa). Across all post designs, the bond strength was greatest in the coronal third and decreased progressively toward the apical third. No significant interaction was observed between post design and root section, indicating their independent effects on bond strength. In failure mode analysis, customized posts predominantly failed at the composite–post interface, whereas double posts mainly exhibited mixed failures, and single posts demonstrated a higher incidence of adhesive failures at the canal walls. From a clinical perspective, double posts may provide a simple and effective approach to improving retention in flared root canals, providing a practical alternative to customized fiber post techniques. Methodologically, the novel 3D-printed model established in this study provides a highly standardized, reproducible platform for the pre-clinical testing of endodontic restorative materials without the confounding anatomical variability of natural teeth.

## Figures and Tables

**Table 1:** Materials used in this study.

Material	Description	Composition	Manufacturer
Freeprint^®^ Crown	Light-Curing Resin	Alkoxylated bisphenol A dimethacrylate, difunctional (meth)acrylate monomers, phosphine oxide-type photoinitiators, and silanized dental glass. Inorganic fillers (size 1.5 μm): 20–40 wt%.	Detax GmbH, Carl-Zeiss-Str. 4, Ettlingen, Germany
TiaDent^TM^ Pillar Fiber Post	Glass Fiber-Reinforced Composite Post Size 2: 1.6 mm Size 3: 1.3 mm	Glass fiber-reinforced composite post made of radiopaque, translucent glass fibers embedded in resin.	Tiadent Richmond Ave, Ste 242, TX, USA
Filtek^TM^ Z250	Universal Restorative Composite	Matrix: Bis-GMA, Bis-EMA, UDMA, TEGDMA. Filler: zirconia, silica (0.01–3.5 μm), 78 wt%, 60 vol%.	3M ESPE 2501 Hudson Rd, St Paul, MN, USA.
Rely X^TM^ U200	Self-Adhesive Resin Cement	Base: methacrylate monomers containing phosphoric acid groups, methacrylate monomers, initiators, stabilizers, and rheological additives. Catalyst: methacrylate monomers, alkaline fillers, silanated fillers, initiator components, stabilizers, pigments, and rheological additives. Zirconia/silica fillers.	3M ESPE 2501 Hudson Rd, St Paul, MN, USA.

Bis-GMA, bisphenol A glycidyl dimethacrylate; Bis-EMA, ethoxylated bisphenol A dimethacrylate; UDMA, urethane dimethacrylate; TEGDMA, triethylene glycol dimethacrylate.

**Table 2:** Descriptive and statistical analyses.

Groups Post Design, Root Section	Push-Out Bond Strength (MPa) Mean ± SD	95% Confidence Interval		Two-Way ANOVA *p*-Value			
		Upper 95%	Lower 95%	Overall Model	Post Design	Root Section	Post Design * Root Design
Double Posts, Coronal	8.26 ± 1.54 ^A^	7.41	9.12	0.044 *	0.027 *	0.0252 *	0.823
Double Posts, Middle	7.77 ± 1.73 ^AB^	6.81	8.73				
Custom Post, Coronal	7.04 ± 1.14 ^BC^	6.41	7.68				
Single Post, Coronal	7.00 ± 1.63 ^BC^	6.10	7.90				
Single Post, Middle	6.86 ± 1.87 ^BC^	5.83	7.89				
Custom Post, Middle	6.82 ± 1.16 ^BC^	6.18	7.46				
Double Posts, Apical	6.71 ± 2.59 ^BC^	5.28	8.14				
Custom Post, Apical	6.41 ± 1.14 ^C^	5.78	7.05				
Single Post, Apical	6.33 ± 1.81 ^C^	5.33	7.33				

Groups connected with similar letters indicate no statistically significant difference (*p* < 0.05). * Statistically significant (*p* < 0.05).

**Table 3:** Estimated marginal means by independent variable “post design”.

Level	Push-Out Bond Strength Mean (MPa) ± SD	95% Confidence Interval		One-Way ANOVA *p*-Value
		Lower 95%	Upper 95%	
Double Posts	7.58 ± 2.07 ^A^	6.96	8.20	0.029 *
Custom Post	6.76 ± 1.15 ^B^	6.41	7.10	
Single Post	6.73 ± 1.75 ^B^	6.20	7.26	

Groups connected with similar letters indicate no statistically significant difference (*p* < 0.05). * Statistically significant (*p* < 0.05).

**Table 4:** Estimated marginal means by independent variable “root section”.

Level	Push-Out Bond Strength Mean (MPa) ± SD	95% Confidence Interval		One-Way ANOVA *p*-Value
		Lower 95%	Upper 95%	
Coronal	7.44 ± 1.54 ^A^	6.97	7.90	0.027 *
Middle	7.15 ± 1.64 ^AB^	6.66	7.64	
Apical	6.49 ± 1.90 ^B^	5.91	7.06	

Groups connected with similar letters indicate no statistically significant difference (*p* < 0.05). * Statistically significant (*p* < 0.05).

**Table 5:** Failure mode analysis by post design and root section.

Post Design, Root Section		Failure Modes				Chi-Square Test	*p*-Value
		1	2	3	4		
Custom Post, Apical	Count	1	12	2	0	80.08	<0.0001 *
	Percentage	6.67	80.00	13.33	0		
Custom Post, Coronal	Count	0	6	4	5		
	Percentage	0	40.00	26.67	33.33		
Custom Post, Middle	Count	0	9	6	0		
	Percentage	0	60.00	40	0		
Double Posts, Apical	Count	2	9	2	1		
	Percentage	14.3	64.3	14.3	7.14		
Double Posts, Coronal	Count	0	4	2	9		
	Percentage	0	26.67	13.33	60.00		
Double Posts, Middle	Count	2	6	6	1		
	Percentage	13.33	40.00	40.00	6.67		
Single Post, Apical	Count	7	2	5	1		
	Percentage	46.66	13.33	33.33	6.67		
Single Post, Coronal	Count	1	12	1	1		
	Percentage	6.67	80.00	6.67	6.67		
Single Post, Middle	Count	6	4	5	0		
	Percentage	40.00	26.67	33.33	0		

* Statistically significant (*p* < 0.05).

## Data Availability

The original contributions presented in the study are included in the article; further inquiries can be directed to the corresponding author.
